# Low serum iron is associated with anemia in CKD stage 1–4 patients with normal transferrin saturations

**DOI:** 10.1038/s41598-021-87401-w

**Published:** 2021-04-16

**Authors:** Pei-Hua Yu, Ming-Yen Lin, Yi-Wen Chiu, Jia-Jung Lee, Shang-Jyh Hwang, Chi-Chih Hung, Hung-Chun Chen

**Affiliations:** 1 Division of Nephrology, Department of Internal Medicine, Kaohsiung Medical University Hospital, Kaohsiung Medical University, 100 Tzyou First Road, San-Ming District, Kaohsiung, 807 Taiwan; 2 Faculty of Renal Care, College of Medicine, Kaohsiung Medical University, Kaohsiung, Taiwan; 3 Regenerative Medicine and Cell Therapy Research Center, Kaohsiung Medical University, Kaohsiung, Taiwan; 4 Graduate Institute of Clinical Medicine, College of Medicine, Kaohsiung Medical University, Kaohsiung, Taiwan

**Keywords:** Medical research, Nephrology

## Abstract

Low transferrin saturation (TSAT), calculated by serum iron divided by total iron-binding capacity (TIBC), indicates iron deficiency. Because malnutrition and inflammation are associated with low TIBC in chronic kidney disease (CKD), TSAT might not reflect iron status or risk for anemia. We examined whether low serum iron was a risk factor for anemia in CKD patients with normal TSAT. Thus we compare the risk for anemia in 2500 CKD stage 1–4 patients divided by TSAT (cutoff: 20%) and serum iron (cutoff: 70 μg/dL in men, 60 μg/dL in women). Our results confirmed low TIBC (< 200 μg/dL) was associated with hypoalbuminemia and high C-reactive protein. In fully-adjusted logistic regression, both “normal TSAT low iron” and “low TSAT low iron” groups were associated with baseline anemia (hemoglobin < 11 g/dL) (odds ratios (OR) 1.56; 95% confidence interval (CI) 1.13–2.16 and OR 2.36; 95% CI 1.76–3.18, respectively) compared with the reference group (normal TSAT normal iron). Sensitivity tests with different cutoffs for TSAT and iron also showed similar results. In patients without anemia, both groups were associated with anemia after 1 year (OR 1.69; 95% CI 1.00–2.83 and OR 1.94; 95% CI 1.11–3.40, respectively). In conclusion, CKD stage 1–4 patients with normal TSAT but low serum iron are still at risk for anemia.

## Introduction

Anemia is a common comorbidity in patients with chronic kidney disease (CKD)^1^. It is defined as hemoglobin (Hb) concentration < 13 g/dL in men and < 12 g/dL in women according to World Health Organization (WHO) criteria^2^. The prevalence of anemia defined by WHO criteria varies geographically from 15.4%, 45.0 to 51.5% in the United States, South Korea and China respectively^3–5^. The severity of anemia is associated with cardiovascular risk, hospitalization, all-cause mortality, and decreased health-related quality of life^6–9^. Underproduction of endogenous erythropoietin by atrophic renal cortex is one of the major causes of anemia in CKD patients, and treatment with erythropoiesis-stimulating agents (ESAs) increases Hb effectively^1^. However, the use of high dose ESAs is associated with adverse events, such as vascular access thromboembolism, stroke, and tumor progression^10,11^.

Iron deficiency is also common in CKD and is another substantial mechanism of anemia in CKD. It is caused by impaired dietary iron absorption, uremic bleeding, and insufficient iron replenishment for Hb synthesis^1^. There are two forms of iron deficiency recognized. One is absolute iron deficiency, and the other is functional iron deficiency. Absolute iron deficiency is defined by severely decreased or absent iron storage in bone marrow, whereas functional iron deficiency is characterized by adequate or increased total body iron stores that are not available to provide sufficient iron to erythroid precursors for erythropoiesis^12^. Iron therapy might be useful for correction of anemia in CKD patients with absolute or functional iron deficiency. In addition, it could lessen the dosage of ESAs in CKD patients^13^.

The definition of iron deficiency in CKD is not clear. The transferrin saturation (TSAT) and serum ferritin levels have limited sensitivity and specificity in patients with CKD for prediction of bone marrow iron stores and the response to iron therapy^14,15^. The Kidney Disease Outcomes Quality Initiative (K/DOQI) 2006 guidelines recommend iron therapy to maintain ferritin > 100 ng/mL and TSAT > 20% in non-dialysis chronic kidney disease (ND-CKD) patients^16^, while the Kidney Disease Improving Global Outcomes (KDIGO) 2012 guidelines recommend iron therapy in ND-CKD patients with TSAT < 30% and serum ferritin < 500 ng/mL if an increase in Hb levels is desired^17^.

However, TSAT, calculated by serum iron divided by total iron-binding capacity (TIBC), could overestimate iron availability in the circulation if TIBC is low. Low transferrin, which is statistically correlated with TIBC^18^, is not uncommon in CKD patients owing to malnutrition and inflammation, and proteinuria^18–21^. A gold standard for iron deficiency, defined as TSAT < 15% and Ferritin < 15 ng/mL in general population^22^, is difficult to be applied to CKD patients with routine laboratory measurements. Moreover, iron deficiency is but one contributor to anemia in this population. Nonetheless, serum iron levels may be useful in reflecting iron availability and utilization in hemoglobin production. A recent review suggested that serum iron may provide more information to guide iron therapy than TSAT^23^. We thus compared the risk for anemia in CKD stage 1–4 patients with low TSAT or with low serum iron. The aim of our study was to investigate additional indications of iron therapy in CKD patients who could benefit from iron therapy aside from current guidelines.

## Methods

### Study participants

This prospective, observational study was conducted in two affiliated hospitals (Kaohsiung Medical University Hospital and Kaohsiung Municipal Hsiao-Kang Hospital) of Kaohsiung Medical University in southern Taiwan. Totally, 3749 patients who joined the Integrated CKD Care Program Kaohsiung for Delaying Dialysis from 11 November, 2002 to 31 May, 2009 were included. CKD was staged according to the definition of K/DOQI guidelines^24^, and the estimated glomerular filtration rate (eGFR) was calculated using the equation of the 4-variable Modification of Diet in Renal Disease (MDRD) study. We excluded patients suffering from CKD stage 5 or those who were lost to follow-up in less than three months; therefore, the final study population was composed of 2500 CKD stage 1–4 patients. All participants in our study provided informed consent to participate. The protocol in the study was approved the Institutional Review Board of Kaohsiung Medical University Hospital. We performed the methods according to the approved guidelines.

To study the impact of TSAT and iron on anemia at baseline, we divided these 2500 CKD patients into four groups—“normal TSAT normal iron”, “normal TSAT low iron”, “low TSAT normal iron”, and “low TSAT low iron”. The cutoff value of TSAT was 20%, while the cutoff values of serum iron were 70 μg/dL in men and 60 μg/dL in women. To study the impact of TSAT and iron level on anemia after 1 year of follow-up, we retrospectively selected 1621 patients without anemia (Hb ≧ 11 g/dL) at baseline, after excluding patients with dialysis, mortality, loss of follow-up, and < 3 hemoglobin measurements in 1 year.

### Data collection

Baseline variables such as demographic features (age and sex), medical history (diabetes mellitus [DM], hypertension, hyperuricemia, and cardiovascular disease [CVD]), clinical parameters (body mass index [BMI] and mean arterial blood pressure [MAP]), and laboratory data (serum creatinine, hemoglobin, albumin, alanine aminotransferase [ALT], white blood cell [WBC], C-reactive protein [CRP], phosphorus, calcium, bicarbonate, uric acid, total cholesterol, triglyceride, glycosylated hemoglobin [HbA1c], and urine protein to creatinine ratio [UPCR]) were collected. The demographic features, medical history and clinical parameters were obtained from medical records and interviews with patients at enrollment. The medical history was obtained by chart review by 2–3 nephrologists. DM and hypertension were defined by clinical diagnosis. Hyperuricemia was defined as uric acid > 7.2 mg/dL in men, > 6.5 mg/dL in women, or under urate-lowering therapy. CVD was defined as clinical diagnosis of ischemic heart disease, congestive heart disease, and cerebrovascular disease. The definition of metabolic syndrome was the presence of three or more of the following five criteria proposed by the Health Promotion Administration, Ministry of Health and Welfare of Taiwan at 2007 (1) fasting blood glucose ≥ 100 mg/dL or diabetes mellitus; (2) systolic blood pressure ≥ 130 mmHg or diastolic blood pressure ≥ 85 mmHg or hypertension; (3) HDL cholesterol > 40 mg/dL in men or > 50 mg/dL in women; (4) triglycerides ≥ 150 mg/dL; and (5) waist circumference ≥ 90 cm in men or ≥ 80 cm in women^25^.

The MAP was calculated by using the formula one-third averaged systolic blood pressure plus two-thirds averaged diastolic blood pressure measured 3 months before and after the enrollment. BMI was calculated as the baseline enrolled measurement by using the formula weight in kilograms divided by height in meters squared. High sensitivity CRP was measured in serum using near the near-infrared particle immunoassay rate method by Beckman Coulter UniCel-DxC 800 (Beckman Coulter); ferritin was measured in serum using two-site immunoenzymatic assay by Beckman Coulter UniCel-DxI 800 (Beckman Coulter); while iron was measured in serum using Fe reagent via timed-endpoint method by Beckman Coulter UniCel-DxC 800 (Beckman Coulter). Transferrin was measured in serum using transferrin reagent via the turbidimetric method by Beckman Coulter UniCel-DxC 800 (Beckman Coulter). And the TIBC value was gained by multiplying transferrin (mg/dL) by a coefficient of 1.4. Laboratory data were averaged and analyzed three months before and after enrollment of the CKD care system.

Malnutrition-inflammation score (MIS) was first proposed by Professor Kalantar-Zadeh for dialysis patients^26^. MIS, modified for CKD patients, has 10 components including body weight change, dietary intake, gastrointestinal symptoms, functional capacity, comorbidity, fat stores, muscle wasting, BMI, albumin, and total iron-binding capacity. Each score component is classified according to 4 levels of severity, from 0 (normal) to 3 (severely abnormal). Measurements were based on Subjective Global Assessment. Malnutrition-inflammation was defined by MIS > 4, based on ROC curve for outcome prediction^27^.

### Outcomes

The main study outcome was anemia at baseline and after 1 year. Anemia was defined as Hb < 11 g/dL as the definition in the study of Trial to Reduce Cardiovascular Events With Aranesp Therapy (TREAT)^28^. We further defined more severe anemia as Hb < 10 g/dL since ESAs therapy was usually started below this level by current clinical practice^29^. Hemoglobin at 1 year was determined by simple linear regression with all hemoglobin measurements in this 1 year.

### Statistical analysis

Statistical results of baseline characteristics of the subjects in the four groups were expressed as mean ± SD for continuous variables with normal distribution, median (25th, 75th percentile) for continuous variables with skewed distribution, and percentages for categorical data.

The significance of differences for normally-distributed continuous variables among groups was tested using one-way ANOVA analysis with post hoc analysis by Fisher’s least significant difference test. And the difference for non-normally-distributed continuous variables was tested using Kruskall–Wallis analysis with post hoc analysis by Dunn–Bonferroni method. The difference in the distribution of categorical variables among groups was tested using the Chi-square test with post hoc analysis by Bonferroni method. Multivariable logistic regression models were used to identify the factors associated with anemia or low TIBC. The adjusted hierarchical covariates were as follows: model (1) demographic factors; model (2) plus comorbidities factors; and model (3) plus other covariates such as albumin, CRP log, ferritin log, and phosphorus. Statistical analyses were conducted using SPSS 21.0 for Windows (SPSS Inc., Chicago, Illinois). Statistical significance was set at a two-sided p-value of < 0.05.

To assess the robustness of our findings, we performed a series of sensitivity analyses, including of: (1) redefining the result of low iron as < 65 μg/dL in men and 50 μg/dL in women, according to the cutoff in our hospital; (2) redefining TSAT < 30%; (3) separating the two “low iron” groups into four subgroups according to serum ferritin levels (“low ferritin” as ferritin < 200 ng/mL, and “high ferritin” as ferritin ≧ 200 ng/mL).

## Results

### Characteristics of patients

The baseline demography, medical history, clinical and laboratory parameters of study participants are shown in Table 1. The mean age (± SD) of patients was 62.4 ± 14.5 years, 64% were men, and the median of eGFR was 35.2 mL/min/1.73 m^2^ (interquartile range [IQR] 24.5–48.7 mL/min/1.73 m^2^). There were 1230 patients (49.2%) suffering from DM, 1513 (60.5%) had hypertension, and 553 (22.1%) had CVD. The mean Hb of patients was 12.2 ± 2.2 g/dL. The subjects in the group of “normal TSAT low iron” were more likely to be older and male, and they also tended to have cerebrovascular disease, lower BMI, higher MIS, lower eGFR and higher UPCR. The post hoc analysis shown in Table 1 disclosed that the two groups of “low iron” status, i.e. the groups of “normal TSAT low iron” and “low TSAT low iron” were considerably correlated with decreased Hb, elevated WBC, elevated CRP levels, and declined albumin levels. Interestingly, the patients with “low TSAT” had higher percentage of female, diabetes and metabolic syndrome. However, only “low TSAT normal iron” group had lower percentage of malnutrition-inflammation and did not have decreased Hb level compared with reference group (normal TSAT normal iron group).

**Table 1:** Characteristics of CKD patients divided by TSAT and serum iron.

Variable	All	Normal TSAT		Low TSAT		*P* value
		Normal iron	Low iron	Normal iron	Low iron	
TSAT (%)		≧ 20	≧ 20	< 20	< 20	
Serum iron female (μg/dL)		≧ 60	< 60	≧ 60	< 60	
Serum iron male (μg/dL)		≧ 70	< 70	≧ 70	< 70	
Number of patients	2500	1451	406	69	574	
**Demographics, medical history, and clinical parameters**						
Age (year)	62.4 (14.5)	61.8 (14.7)	65.1 (13.4)*	63.2 (12.6)	61.9 (14.7)	< 0.001
Sex (female) (%)	899 (36.0%)	467 (32.2%)	118 (29.1%)	48 (69.6%)*	266 (46.3%)*	< 0.001
Diabetes mellitus (%)	1230 (49.2%)	617 (42.5%)	225 (55.4%)*	42 (60.9%)*	346 (60.3%)*	< 0.001
Cardiovascular disease (%)	553 (22.1%)	287 (19.8%)	106 (26.1%)*	12 (17.4%)*	148 (25.8%)*	0.003
Hypertension (%)	1513 (60.5%)	863 (59.5%)	245 (60.3%)	47 (68.1%)	358 (62.4%)	0.368
Hyperuricemia (%)	448 (17.9%)	288 (19.8%)	74 (18.2%)	8 (11.6%)*	78 (13.6%)*	0.005
Metabolic syndrome (%)	1673 (66.9%)	941 (64.9%)	259 (63.8%)	58 (84.1%)*	415 (72.3%)*	< 0.001
Malnutrition-inflammation (%)	1159 (46.4%)	553 (38.1%)	285 (70.2%)*	18 (26.1%)*	303 (52.8%)*	< 0.001
MAP	99.4 (13.4)	99.2 (13.1)	99.0 (14.4)	97.7 (11.7)	100.3 (13.6)	0.225
BMI	25.1 (4.0)	25.2 (3.8)	24.6 (4.1)	26.3 (4.2)	25.1 (4.4)	0.004
**Renal function status**						
eGFR (ml/min/1.73 m^2^)	35.2 (24.5–48.7)	36.7 (26.1–49.7)	28.9 (20.7–41.3)*	37.0 (27.0–52.8)	32.9 (22.6–46.2)	< 0.001
UPCR (mg/g)	691.8 (247–1797)	585 (217–1446)	1165 (354–2899)	675 (195–1479)	848 (281–2330)	< 0.001
**Laboratory data**						
Hemoglobin (g/dL)	12.2 (2.2)	12.7 (2.1)	11.4 (2.1)*	12.3 (1.9)	11.5 (2.2)*	< 0.001
Albumin (g/dL)	3.9 (0.5)	4.0 (0.5)	3.7 (0.7)*	4.1 (0.4)	3.8 (0.5)*	< 0.001
ALT (mg/dL)	26.9 (24.4)	28.0 (26.1)	24.8 (25.4)*	35.5 (29.8)*	24.6 (17.6)*	< 0.001
WBC (× 1,000 cells/μl)	7.2 (2.3)	6.9 (2.1)	7.5 (2.3)*	7.0 (2.5)	7.6 (2.5)*	< 0.001
CRP (mg/l)	1.0 (0.3–4.7)	0.8 (0.3–3.1)	1.7 (0.5–8.1)*	0.5 (0.2–2.3)	1.4 (0.4–7.0)*	< 0.001
Phosphorus (mg/dL)	3.9 (0.8)	3.8 (0.8)	4.0 (0.9)*	3.9 (0.6)	4.0 (0.9)*	0.003
Calcium (mg/dL)	9.2 (0.7)	9.3 (0.6)	9.1 (0.8)*	9.4 (0.6)*	9.3 (0.7)	< 0.001
Bicarbonate (mEq/L)	23.7 (3.7)	24.0 (3.6)	22.9 (4.0)*	24.4 (3.2)	23.6 (3.9)*	< 0.001
Uric acid (mg/dL)	7.6 (1.9)	7.6 (1.9)	7.8 (1.9)	7.2 (1.7)	7.5 (2.1)	0.074
Total cholesterol (mg/dL)	194 (166–225)	193 (167–223)	192 (163–222)	190 (167–223)	197 (165–232)*	0.397
Triglyceride (mg/dL)	127 (92–189)	126 (92–183)	124 (91–181)	165 (90–223)*	133 (95–208)	0.032
HbA1c (%)	6.7 (1.7)	6.4 (1.5)	6.7 (1.8)*	7.0 (1.7)*	7.1 (2.0)*	< 0.001

Data were presented as mean (standard error), median (interquartile range), or count (percentage). *P* value column meant differences among groups (< 0.05 was significant).

*Meant significant difference (P < 0.05) compared with normal TSAT normal iron group in post hoc analysis. The significance of differences for normally-distributed continuous variables among groups was tested using one-way ANOVA with post hoc analysis by Fisher’s least significant difference test and, for non-normally-distributed continuous variables, it was tested using Kruskall–Wallis analysis with post hoc analysis by Dunn–Bonferroni method. The difference in the distribution of categorical variables among groups was tested using the Chi-square test with post hoc analysis by Bonferroni method.

### Iron status and malnutrition-inflammation

The iron status of participants is shown in Table 2. The average TSAT was 30.4 ± 17.8%, the mean serum iron was 77.8 ± 36.8 μg/dL, the average TIBC was 279.2 ± 80.2 μg/dL, and the median of ferritin was 179 ng/ml (IQR 96–322 ng/mL). A low percentage (0.8%) of patients received iron treatment. ESAs were not applied in our study population because there’s no reimbursement by national health insurance. The levels of hemoglobin, TSAT, TIBC and ferritin by gender were shown on Supplementary Fig. S1. Hemoglobin and TIBC were lower in CKD stage 4, but TSAT in women with CKD stage 4 was not lower.

**Table 2:** Iron status divided by TSAT and serum iron in CKD 1–4 patients.

Variable	All	Normal TSAT		Low TSAT		*P* value
		Normal iron	Low iron	Normal iron	Low iron	
TSAT (%)	30.4 (17.8)	38.1 (17.2)	30.0 (13.2)*	18.2 (1.6)*	13.0 (5.4)*	< 0.001
Iron (μg/dL)	77.8 (36.8)	100.4 (29.9)	54.8 (12.2)*	71.5 (8.2)*	38.5 (17.1)*	< 0.001
TIBC (μg/dL)	279.2 (80.2)	283.3 (68.5)	208.3 (73.1)*	395.6 (52.8)*	304.1 (78.9)	< 0.001
Ferritin (ng/ml)	179 (96–322)	200 (114–338)	241 (135–427)*	71 (27–148)*	112 (49–234)*	< 0.001

Data were presented as mean (standard error), median (interquartile range), or count (percentage). *P* value column meant differences among groups (< 0.05 was significant).

*Meant significant difference (P < 0.05) compared with normal TSAT normal iron group in post hoc analysis. The significance of differences for normally-distributed continuous variables among groups was tested using one-way ANOVA with post hoc analysis by Fisher’s least significant difference test and, for non-normally-distributed continuous variables, it was tested using Kruskall–Wallis analysis with post hoc analysis by Dunn–Bonferroni method. The difference in the distribution of categorical variables among groups was tested using the Chi-square test with post hoc analysis by Bonferroni method.

**Figure 1:**
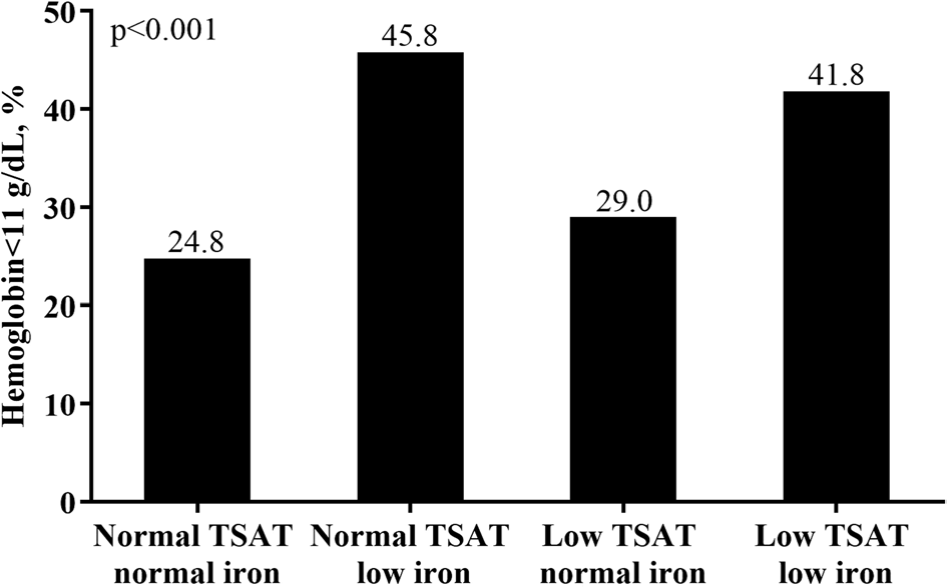
Anemia divided by TSAT and serum iron.

**Figure 2:**
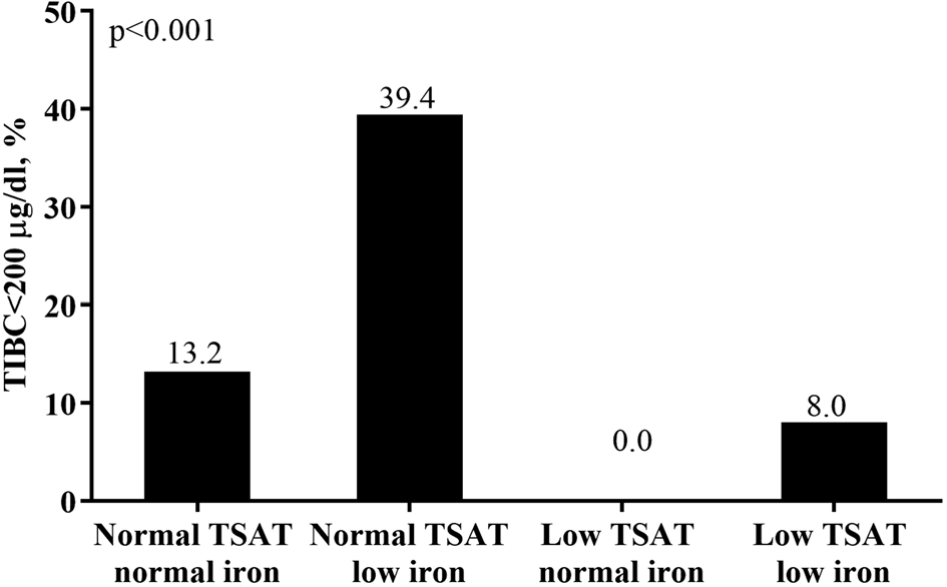
Low TIBC devided by TSAT and serum iron.

**Figure 3:**
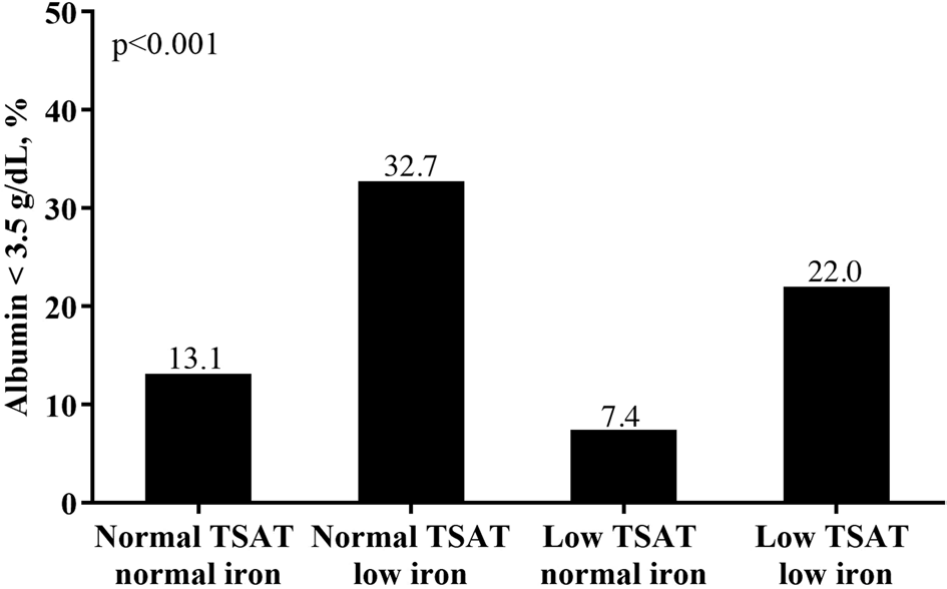
Malnutrition divided by TSAT and serum iron.

**Figure 4:**
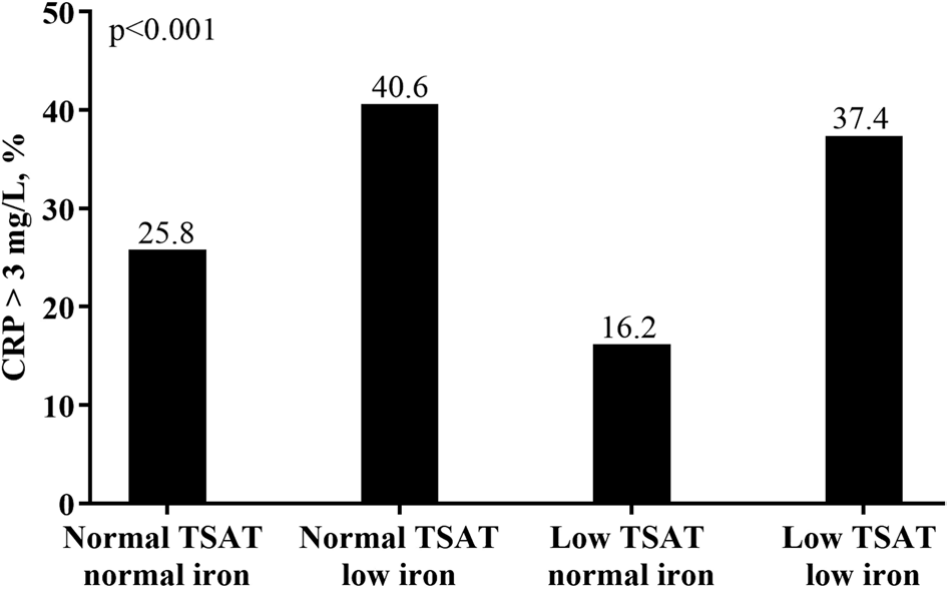
Inflammation divided by TSAT and serum iron.

In Table 2, a considerably higher proportion of patients in the two “low iron” groups suffered from anemia, with 45.8% of patients in the “normal TSAT low iron” group and 41.8% of patients in the “low TSAT low iron” group had Hb < 11 g/dL (Fig. 1). In addition, we found that the subjects in the group of “normal TSAT low iron” had highest percentage of low TIBC (39.4% with TIBC < 200 μg/dL) (Fig. 2), as well as the highest percentage of hypoalbuminemia (32.7% with Albumin < 3.5 g/dL) (Fig. 3) and elevated CRP levels (40.6% with CRP > 3 mg/L) (Fig. 4).

### Factors associated with low TIBC

As the result of the multivariable logistic regression analysis depicts in Table 3, low TIBC (< 200 μg/dL) was significantly associated with low albumin (OR 0.56; 95% CI 0.49 to 0.65; P < 0.001) and high CRP values (OR 1.23; 95% CI 1.08 to 1.40; P = 0.002). Additionally, we also found that the subjects with lower BMI, lower iron, higher ferritin, and lower phosphorus levels were prone to have low TIBC.

**Table 3:** Multivariable logistic regression (fully-adjusted model) for low TIBC (< 200 μg/dL).

Variable	Odds ratio	95% CI	P value
Hemoglobin (per 1 SD)	0.87	0.73 to 1.04	0.118
Age (per 1 SD)	0.91	0.79 to 1.04	0.174
Female (vs male)	1.63	1.22 to 2.18	0.001
eGFR (per 1SD)	1.54	1.33 to 1.79	< 0.001
log UPCR (per 1 SD)	1.03	0.88 to 1.20	0.755
Diabetes mellitus	0.74	0.56 to 0.98	0.033
Cardiovascular disease	1.00	0.73 to 1.37	0.989
Severe liver disease	1.06	0.59 to 1.90	0.848
Cancer	0.80	0.49 to 1.32	0.384
Mean BP (per 1 SD)	0.94	0.82 to 1.07	0.322
Body mass index (per 1 SD)	0.80	0.70 to 0.92	0.001
Albumin (per 1 SD)	0.56	0.49 to 0.65	< 0.001
log CRP (per 1 SD)	1.23	1.08 to 1.40	0.002
Phosphorus (per 1 SD)	0.83	0.73 to 0.96	0.009
log Ferritin (per 1 SD)	2.70	2.29 to 3.19	< 0.001
Iron (per 1 SD)	0.70	0.60 to 0.80	< 0.001

*Log* log-transformation.

### Association of TSAT, serum iron and ferritin with baseline anemia

As compared with the subjects in the “normal TSAT normal iron” group, the subjects in both “low iron” groups had significantly higher OR for Hb < 11 g/dL (normal TSAT low iron: OR 2.44; 95% CI 1.94–3.07; low TSAT low iron: OR 2.22; 95% CI 1.81–2.72) and for Hb < 10 g/dL (normal TSAT low iron: OR 2.78; 95% CI 2.12–3.66; low TSAT low iron: OR 2.86; 95% CI 2.25–3.64) (Table 4). After adjustment for demography, comorbidity, or other covariates, the results remained the similar (Table 4). Moreover, we further conducted sensitivity tests with different definitions of iron (Supplementary Table S1) and TSAT (Supplementary Table S2), and similar results were shown. Furthermore, we divided the two groups of “low iron” into four subgroups by ferritin levels (Supplementary Table S3), and it still showed that anemia was relevant to low iron regardless of the ferritin levels.

**Table 4:** Association of TSAT and serum iron with anemia.

Variable	Normal TSAT		Low TSAT	
	Normal iron	Low iron	Normal iron	Low iron
**Odds ratio for Hb < 11 g/dL**				
Unadjusted	1 (reference)	2.44 (1.94–3.07)*	1.17 (0.68–2.02)	2.22 (1.81–2.72)*
Model 1	1 (reference)	1.95 (1.49–2.54)*	0.78 (0.41–1.46)	1.93 (1.52–2.44)*
Model 2	1 (reference)	1.91 (1.46–2.50)*	0.74 (0.39–1.41)	1.95 (1.54–2.48)*
Model 3	1 (reference)	1.54 (1.16–2.05)*	0.91 (0.48–1.74)	1.79 (1.38–2.32)*
**Odds ratio for Hb < 10 g/dL**				
Unadjusted	1 (reference)	2.78 (2.12–3.66)*	1.01 (0.47–2.14)	2.86 (2.25–3.64)*
Model 1	1 (reference)	1.99 (1.47–2.70)*	0.72 (0.32–1.63)	2.45 (1.88–3.21)*
Model 2	1 (reference)	1.99 (1.46–2.71)*	0.71 (0.31–1.63)	2.56 (1.95–3.37)*
Model 3	1 (reference)	1.56 (1.13–2.16)*	0.97 (0.42–2.23)	2.36 (1.76–3.18)*

Model 1 adjusts for Age, Gender, eGFR, UPCR log.

Model 2 adjusts for covariates in model 1 plus Diabetes mellitus, Cardiovascular disease, Severe liver disease, Cancer, Mean BP, and Body mass index.

Model 3 adjusts for covariates in model 2 plus Albumin, CRP log, Ferritin Log, and Phosphorus.

*Meant statistically significant compared with reference group.

### Association of TSAT, and serum iron with anemia after 1 year in patients without anemia

We followed 1695 patients with hemoglobin ≧ 11 g/dL at baseline and 22/1091, 9/220, 0/50 and 9/334 patients encountered dialysis or mortality in “normal TSAT normal iron”, “normal TSAT low iron”, “low TSAT normal iron” and “low TSAT low iron” groups, respectively, in the first year of follow-up. After further excluding those loss of follow-up, and < 3 hemoglobin measurements, 1621 patients without anemia entered the analysis. 164 of them had Hb < 11 g/dL after 1 year. The subjects in both “low iron” groups had significantly higher OR for Hb < 11 g/dL after 1 year (normal TSAT low iron: OR 1.69; 95% CI 1.00–2.83; low TSAT low iron: OR 1.94; 95% CI 1.11–3.40), after adjustment for baseline hemoglobin and other variables in Tables 4 and 5.

**Table 5:** Association of TSAT and serum iron with anemia after 1 year in patients without anemia.

Variable	Normal TSAT		Low TSAT	
	Normal iron	Low iron	Normal iron	Low iron
Number of patients	1040	202	50	329
**Odds ratio for Hb < 11 g/dL after 1 year**				
Unadjusted	1 (reference)	2.48 (1.69–3.66)*	1.89 (0.78–4.59)	3.21 (2.09–4.93)*
Model 1	1 (reference)	2.42 (1.58–3.71)*	1.20 (0.44–3.23)	2.76 (1.72–4.44)*
Model 2	1 (reference)	2.49 (1.62–3.85)*	1.22 (0.45–3.31)	2.76 (1.71–4.45)*
Model 3	1 (reference)	1.69 (1.00–2.83)*	1.43 (0.50–4.62)	1.94 (1.11–3.40)*

Model 1 adjusts for Age, Gender, eGFR, UPCR log.

Model 2 adjusts for covariates in model 1 plus Diabetes mellitus, Cardiovascular disease, Severe liver disease, Cancer, Mean BP, and Body mass index.

Model 3 adjusts for covariates in model 2 plus baseline Hemoglobin, Albumin, CRP log, Ferritin Log, and Phosphorus.

*Meant statistically significant compared with reference group.

## Discussion

In our observational study, CKD stage 1–4 patients with low serum iron still had the risk of anemia no matter what the TSAT levels. Notably, the subjects of the “normal TSAT low iron” group had the highest percentage of anemia, malnutrition, and inflammation. Furthermore, we also discovered that low TIBC was significantly associated with malnutrition and inflammation. The possible explanation of these 4 groups was presented in Supplementary Table S4.

The first important finding of our study was identification of low iron value as an independent risk factor of anemia in CKD stage 1–4 patients regardless of TSAT levels. Iron deficiency anemia, defined as TSAT < 15% and Ferritin < 15 ng/mL in the general population^22^, is quite different in CKD groups since the majority of CKD patients suffer from functional iron deficiency rather than absolute iron deficiency^30^. Although current guidelines use TSAT and serum ferritin as the standard tools for stratifying CKD patients who will/will not respond to iron therapy, there are still some limitations. For example, serum ferritin concentration may increase in inflammation, malignancy, and liver disease^31^. Therefore, other iron indices such as reticulocyte Hb content (CHr), percentage of hypochromic red blood cell (PHRC), soluble transferrin receptor test (sTfR), and hepcidin have all been utilized to evaluate iron metabolism in CKD patients^32–36^. Hepcidin, which regulates the dietary iron absorption from gut and the release of iron from macrophages, can help us to clarify the capacity of iron availability of the patients^37^. sTfR can reflects erythropoietic activity and is not affected by inflammation^36,37^. However, it cannot reflect iron availability completely^38^. Instead, the ratio of sTfR to log_10_ferritin is superior to sTfR alone in differentiating iron deficiency anemia from anemia of chronic disease^39^. CHr and PHRC reflect iron availability to the bone marrow and iron-restricted erythropoiesis^32,37^. All of these biomarkers can help us to evaluate the response to iron therapy especially for intravenous iron^36^. However, the cost of techniques and the availability of extensive testing must be concerned, whereas in contrast, serum iron is inexpensive and used widely. Our study suggests that low serum iron value is independently associated with anemia in CKD group. To our knowledge, this is the first paper to utilize serum iron to predict anemia in CKD subjects.

The second important finding of our study is that the subjects of the “normal TSAT low iron” group had the highest percentage of anemia, malnutrition, and inflammation. We further interpret the characteristics of the patients in this group as “anemia of chronic inflammation”. Among them, low TIBC is an important associated factor for malnutrition and inflammation. Transferrin, a 90-kDa beta-globulin, primarily synthesized by the liver^19^, has a high statistical correlation with TIBC^18^. Kalantar-Zadeh et al. indicated that low serum TIBC is related to protein-energy wasting, inflammation, poor quality of life, and mortality^19^. They found that TIBC is positively associated with nutritional biomarkers such as serum albumin and negatively associated with several inflammatory markers such as log CRP. Our data shown in Table 3 reveals similar results. These findings might explain our observation that subjects with “normal TSAT low iron” have highest percentage of hypoalbuminemia and elevated CRP levels as depicted in Figs. 3 and 4, respectively. Since the percentage of low TIBC value is highest in this group, it is reasonable to infer that the malnutrition and inflammation in this group are correlated with low TIBC. Additionally, our multivariable logistic regression analysis shows that TIBC has positive correlation with serum iron and negative correlation with serum ferritin. This result is compatible with the result proved by Kalantar-Zadeh et al.^19,40^, who found that serum iron is low while serum ferritin is high in poorly nourished groups. Indeed, they also recommended not to use TSAT as a diagnostic tool of iron deficiency if serum TIBC is less than 200 mg/dL. They believed that TIBC, as the denominator of iron saturation ratio, might mistakenly lead TSAT ratio to normal or increased ranges especially in malnourished patients of whom the TIBC value is decreased^40^. Since the highest percentage of hypoalbuminemia and low TIBC value in the group of “normal TSAT low iron” was shown by our findings, it is inferred that the TSAT in this group is erroneously high due to low TIBC levels caused by malnutrition. By comparison, from this group, it appears that low serum iron is more associated with anemia in CKD patients. Our study cannot determine if iron replacement or supplementation in patients with low serum iron and normal transferrin saturations will be beneficial. This combination of findings should at the very least prompt further scrutiny of nutritional and other factors that may affect the TIBC in CKD patients.

Similarly, the concept of low TIBC caused by malnutrition and inflammation could be utilized to explain the subjects in “low TSAT low iron” group, who was interpreted as “iron deficiency combined with inflammation”. Unlike the patients with absolute iron deficiency anemia, the subjects in “low TSAT low iron” group have higher malnutrition-inflammation, ferritin and CRP levels and lower TIBC values. These findings might be partially explained by inflammation.

“Low TSAT normal iron” group had lower percentage of malnutrition-inflammation, although higher percentage of metabolic syndrome. Our previous study had shown an obesity paradox in CKD population that high BMI was not associated with all-cause mortality, especially in female^41^. Our new analysis of malnutrition-inflammation score may explain the obesity paradox in CKD population^27^. In addition, none of patients with “low TSAT normal iron” and hemoglobin ≧ 11 g/dL encountered dialysis or mortality in the 1st year, which showed the best survival, compared with the other groups. We hypothesized that the stability of serum iron levels was driven by reductions in hepcidin levels. The low hepcidin levels would improve the iron absorption from gut and the iron release from macrophage, which induced low ferritin levels. Besides, an increase in TIBC also improved iron delivery to the bone marrow. Thus, even if TSAT was low, the increased iron bioavailability allowed adequate erythropoiesis. According to the above characteristics, we interpreted the subjects in “low TSAT normal iron” group as “iron deficiency with greater iron availability (i.e., rapid iron response)”.

There are several limitations in this study. First, the subjects was Taiwanese without iron and EPO treatment, more data in other populations and patients treated with EPO are needed before broader application. Second, we did not include patients of CKD stage 5 without dialysis. In these patients, EPO deficiency and ESAs treatment could be more important. Third, we only got the data of serum iron and transferrin in the beginning of enrollment. This might cause variability and affect clinical decision making of iron therapy. Fourth, only a few patients (0.8%) in our study received iron treatment. Neither causality nor the temporal relationship between serum iron and anemia can be determined with this observational study. Fifth, the longitudinal study about the impact of TSAT and iron on anemia after 1 year was retrospective and did not evaluate many confounders. Finally, we did not examine novel markers of iron homeostasis such as hepcidin, sTfR/log_10_ferritin, PHRC, and CHr, which help to determine iron availability or erythropoiesis activity, and also to predict the response to iron therapy. Especially for hepcidin, it might prove that serum iron is better than TSAT to define anemia in CKD patients with inflammation. Further study is needed to clarify the state of iron deficiency and the capacity of iron availability by using these new iron indices.

## Conclusion

In conclusion, our study suggests that CKD stage 1–4 patients with normal TSAT but low serum iron are still at risk for anemia. “Normal TSAT low iron” group is associated with inflammation and malnutrition, and could be at risk for “anemia of chronic inflammation”. Low serum iron might be a considerable risk factor of anemia in CKD stage 1–4 subjects regardless of TSAT levels. Further research is needed to confirm the effect of iron treatment in CKD stage 1–4 patients with low serum iron value.

## Supplementary Information

Supplementary Information.

## Data Availability

All data generated or analyzed during this study are included in this published article (and its Supplementary Information files).
