# Supplementary material for: Low serum iron is associated with anemia in CKD stage 1–4 patients with normal transferrin saturations
Source: Sci Rep. 2021 Apr 16;11:8343. doi: 10.1038/s41598-021-87401-w (PMC8052429; doi:10.1038/s41598-021-87401-w)
Supplement: Supplementary file 1 — Supplementary Information. [file 41598_2021_87401_MOESM1_ESM.docx]

**CKD stage 1-4 patients with normal transferrin saturation but low serum iron are still at risk for anemia**

Pei-Hua Yu^1,4^, Ming-Yen Lin^1^, Yi-Wen Chiu^1,2^, Jia-Jung Lee^1,3^, Shang-Jyh Hwang^1,2^, Chi-Chih Hung^1,3*^, Hung-Chun Chen^1,2^

1. Division of Nephrology, Department of Internal Medicine, Kaohsiung Medical University Hospital, Kaohsiung Medical University, Kaohsiung, Taiwan
2. Faculty of Renal Care, College of Medicine, Kaohsiung Medical University, Kaohsiung, Taiwan
3. Regenerative Medicine and Cell Therapy Research Center, Kaohsiung Medical University, Kaohsiung, Taiwan
4. Graduate Institute of Clinical Medicine, College of Medicine, Kaohsiung Medical University, Kaohsiung, Taiwan

The two underlined authors equally contributed to this article

Supplementary Table S1. Odds ratios for anemia according to TSAT and iron (men <65μg/dL, women <50μg/dL) groups. Model 1, 2 and 3 are the same as Table 4.

| Variable | Normal TSAT | | |  | Low TSAT | | |
| --- | --- | --- | --- | --- | --- | --- | --- |
|  | Normal iron | Low iron | |  | Normal iron | | Low iron |
| **Odds ratio for Hb <10 g/dL** | | |  |  |  |  | |
| Unadjusted | 1 (reference) | 2.81 (2.07-3.83)* | |  | 1.46 (0.94-2.26) | | 2.71 (2.13-3.46)* |
| Model 1 | 1 (reference) | 2.45 (1.72-3.50)* | |  | 0.97 (0.60-1.58) | | 2.64 (2.01-3.47)* |
| Model 2 | 1 (reference) | 2.43 (1.69-3.49)* | |  | 0.99 (0.60-1.61) | | 2.77 (2.09-3.67)* |
| Model 3 | 1 (reference) | 1.74 (1.18-2.56)* | |  | 1.23 (0.73-2.05) | | 2.45 (1.81-3.32)* |
| **Odds ratio for Hb <11 g/dL** | | |  |  |  |  | |
| Unadjusted | 1 (reference) | 2.24 (1.71-2.94)* | |  | 1.36 (0.96-1.94) | | 2.10 (1.70-2.58)* |
| Model 1 | 1 (reference) | 2.23 (1.62-3.06)* | |  | 0.79 (0.52-1.19) | | 2.13 (1.67-2.72)* |
| Model 2 | 1 (reference) | 2.16 (1.56-2.98)* | |  | 0.77 (0.51-1.16) | | 2.18 (1.70-2.80)* |
| Model 3 | 1 (reference) | 1.61 (1.14-2.27)* | |  | 0.89 (0.58-1.37) | | 1.94 (1.48-2.54)* |

Supplementary Table S2. Odds ratios for anemia according to TSAT (<30%) and iron groups. Model 1, 2 and 3 are the same as Table 4.

| Variable | Normal TSAT | | | |  | | Low TSAT | |
| --- | --- | --- | --- | --- | --- | --- | --- | --- |
|  | Normal iron | Low iron | | |  | | Normal iron | Low iron |
| **Odds ratio for Hb <10 g/dL** | | | | | | | | |
| Unadjusted | 1 (reference) | 3.65 (2.33-5.74)* | | |  | | 1.02 (0.74-1.41) | 2.76 (2.15-3.56)* |
| Model 1 | 1 (reference) | 2.53 (1.51-4.21)* | | |  | | 0.81 (0.57-1.14) | 2.04 (1.55-2.70)* |
| Model 2 | 1 (reference) | 2.59 (1.53-4.38)* | | |  | | 0.82 (0.58-1.17) | 2.11 (1.59-2.80)* |
| Model 3 | 1 (reference) | 1.88 (1.07-3.30)* | | |  | | 0.98 (0.68-1.42) | 1.97 (1.46-2.67)* |
| **Odds ratio for Hb <11 g/dL** | | |  |  | |  | |  |
| Unadjusted | 1 (reference) | 3.17 (2.11-4.77)* | | |  | | 1.36 (1.07-1.72)* | 2.54 (2.07-3.12)* |
| Model 1 | 1 (reference) | 2.39 (1.48-3.84)* | | |  | | 1.04 (0.79-1.36) | 1.94 (1.54-2.46)* |
| Model 2 | 1 (reference) | 2.43 (1.49-3.94)* | | |  | | 1.04 (0.79-1.36) | 1.95 (1.53-2.47)* |
| Model 3 | 1 (reference) | 1.86 (1.10-3.14)* | | |  | | 1.22 (0.92-1.63) | 1.83 (1.42-2.37)* |

Supplementary Table S3. Odds ratios for anemia according to TSAT, iron and Ferritin groups. Model 1, 2 and 3 are the same as Table 4.

| Variable | Normal TSAT | | | | |  | Low TSAT | | |
| --- | --- | --- | --- | --- | --- | --- | --- | --- | --- |
|  | Normal iron | | Low iron | | |  | Normal iron | Low iron | |
|  |  | High Ferritin | | | Low Ferritin |  |  | High Ferritin | Low ferritin |
| **Odds ratio for Hb <10 g/dl** | | | |  | |  |  |  | |
| Unadjusted | 1 (reference) | 3.40 (2.46-4.68)** | | | 2.04 (1.37-3.05)** |  | 1.007 (0.473-2.142) | 3.62 (2.54-5.15)** | 2.57 (1.95-3.38)** |
| Model 1 | 1 (reference) | 2.32 (1.62-3.33)** | | | 1.59 (1.02-2.45)* |  | 0.718 (0.317-1.626) | 2.78 (1.87-4.13)** | 2.31 (1.70-3.13)** |
| Model 2 | 1 (reference) | 2.29 (1.69-3.12)** | | | 1.64 (1.05-2.54)* |  | 0.713 (0.314-1.619) | 2.80 (1.88-4.18)** | 2.29 (1.69-3.12)** |
| Model 3 | 1 (reference) | 1.67 (1.13-2.46)* | | | 1.44 (0.91-2.28) |  | 0.925 (0.404-2.118) | 2.38 (1.55-3.64)** | 2.23 (1.62-3.08)** |
| **Odds ratio for Hb <11 g/dl** | | | |  | |  |  |  | |
| Unadjusted | 1 (reference) | 2.87 (2.16-3.82)** | | | 1.94 (1.39-2.70)** |  | 1.173 (0.682-2.019) | 2.70 (1.96-3.71)** | 2.04 (1.62-2.57)** |
| Model 1 | 1 (reference) | 2.22 (1.60-3.09)** | | | 1.63 (1.12-2.38)* |  | 0.772 (0.411-1.449) | 2.19 (1.51-3.18)** | 1.82 (1.39-2.38)** |
| Model 2 | 1 (reference) | 1.79 (1.37-2.35)** | | | 1.65 (1.13-2.41)* |  | 0.753 (0.399-1.423) | 2.18 (1.50-3.16)** | 1.79 (1.37-2.35)** |
| Model 3 | 1 (reference) | 1.59 (1.12-2.27)* | | | 1.48 (0.99-2.20) |  | 0.889 (0.468-1.691) | 1.83 (1.23-2.72)* | 1.72 (1.29-2.28)** |

Supplementary Table S4. The possible explanation of the 4 groups in our study. ** We considered that TIBC was equivalent to transferrin according to the method of TIBC measurement. #* *Malnutrition- inflammation was defined by malnutrition-inflammation score*

| Variable | Normal TSAT | |  | Low TSAT | |
| --- | --- | --- | --- | --- | --- |
|  | Normal iron | Low iron |  | Normal iron | Low iron |
| **Possible interpretation** | Normal | Anemia of chronic inflammation |  | Iron deficiency with greater iron availability (i.e., rapid iron response) | Iron deficiency + Inflammation |
| Iron | Normal | Low |  | Normal | Low |
| TIBC (Transferrin)* | Normal | Low |  | Elevated | Normal |
| Ferritin | Normal | Normal to elevated |  | Low | Low to normal |
| Malnutrition-inflammation (%)^#^ | Medium | High |  | Low to medium | Medium to high |
| CRP | Normal | Increased |  | Normal | Increased |

Supplementary Figure 1a. Hemoglobin levels of CKD stage 1-4 in male and female





Supplementary Figure 1b. TSAT levels of CKD stage 1-4 in male and female





Supplementary Figure 1c. TIBC levels of CKD stage 1-4 in male and female





Supplementary Figure 1d. Ferritin levels of CKD stage 1-4 in male and female
